# Structural characteristics and policy differences of digital health management policies in China: A quantitative analysis based on the PMC index model

**DOI:** 10.1371/journal.pone.0362220

**Published:** 2026-11-04

**Authors:** Tong Lyu

**Affiliations:** School of Humanities and Arts, China University of Mining and Technology, Xuzhou, China; PLOS, UNITED KINGDOM OF GREAT BRITAIN AND NORTHERN IRELAND

## Abstract

Digital health governance increasingly relies on policy systems that coordinate data resources, information platforms, standards, service delivery, regulation, security, innovation, and implementation support. However, the structural composition and cross-policy heterogeneity of China’s national digital health policy system remain insufficiently quantified. This study develops a domain-specific Policy Modeling Consistency (PMC) framework to evaluate the structural coverage of national-level digital health management policies in China. Using a source-locked corpus covering 2016–2025, 42 eligible policies were identified, of which 39 with complete and auditable texts were included in the quantitative analysis. The framework comprises nine dimensions and 48 binary indicators, generating 1,872 policy-by-indicator coding decisions. An independently coded 11-policy subset yielded 96.78% agreement and a Cohen’s kappa of 0.914. The mean PMC score was 6.654 (SD = 1.456; median = 6.500; range = 3.467–9.000). Under the study’s structural-coverage thresholds, nine policies were classified as perfect, 11 as good, 16 as acceptable, and three as poor. Regulatory governance, standards and institutional arrangements, and data resources showed the highest average coverage, whereas data protection and industrial innovation showed the lowest coverage and the greatest cross-policy variation. Leave-one-policy-out and policy-family sensitivity analyses confirmed the stability of the principal structural pattern. These findings reveal a policy architecture with relatively extensive governance, institutional, and data-related provisions but more uneven protection- and innovation-related coverage. The proposed framework provides a reproducible basis for comparing policy-text structure while avoiding the interpretation of textual completeness as evidence of implementation quality or policy effectiveness.

## 1 Introduction

Recent research links digital-health innovation to universal health coverage and equity [1], examines how digital-health technologies affect regional medical-resource allocation in China [2], documents associations among digital literacy, health literacy, and digital-health behaviors in rural China [3], and analyzes digital-health governance through a whole-of-society lens [4]. Together, this literature shows that digital health spans technological, service, equity, and governance questions. A structured and reproducible comparison is therefore needed to identify what individual policy texts cover and how their configurations differ.

Chinese empirical studies have examined heterogeneous effects of internet use among older adults [5], public satisfaction with implementation of the Healthy China Action Plan [6], and health effects associated with Healthy China 2030 [7]. These studies address users or broad health strategies rather than reproducible comparison of explicit elements across national digital-health policy texts, leaving a distinct policy-structure question for the present study.

Policy scholarship has examined the making and program design of Healthy China 2030 [8] and internet medical policies using topic identification and content analysis [9]. The present study extends this literature by evaluating national-level digital health management policies with source-locked texts, a fully specified 48-indicator codebook, and transparent reliability and robustness procedures. The objective is to characterize structural coverage and cross-policy heterogeneity without treating textual completeness as evidence of implementation quality or real-world effectiveness.

Digital-health scholarship emphasizes that infrastructure foundations matter when technological investment is translated into impact [10]. Recent work has separately reviewed AI and telemedicine in rural communities [11], constraints affecting consent to share electronic-health-record data [12], and integration of environmental data into electronic health records [13]. These studies illustrate the breadth of technological, data, and implementation questions that a policy framework may need to address.

Health-data governance research addresses public engagement in genomics and health-data governance [14], health-data justice [15], Indigenous data-governance approaches [16], contextual integrity for personal health information [17], governance features of health-data hubs [18], organization of a national digital service for personal health data [19], and trust-based governance of health-data research [20]. Together, these strands show why access, legitimacy, institutional responsibility, privacy, and trust must be treated as related but analytically distinct policy elements.

Research relevant to digitally enabled service delivery in China includes studies of rural healthcare access [21], the Trinity smart-hospital construction policy [22], sustainability of health and fitness information-platform ecosystems [23], and service quality in integrated health and social care [24]. Their outcome and domain scopes differ, but they collectively illustrate the range of service, platform, and organizational contexts in which digital-health policies operate.

Complementary studies examine online-pharmacy regulation in China [25], mobile health and telemedicine in rural reproductive healthcare [26], policy trajectories for internet telemedicine [27], and China’s regulatory approaches to health-data protection [28]. This literature provides focused analyses of individual domains; a common structural-coverage framework offers a different, cross-policy comparison of explicit textual elements.

The PMC index has been used to examine the role of traditional medicine in emergency health-system policy [29] and Chinese long-term-care insurance policies [30]. By decomposing policy texts into observable indicators and applying a common coding scheme, PMC studies can compare structural coverage while keeping that construct distinct from implementation or outcomes.

Despite these advances, three gaps remain. First, existing studies tend to examine particular technologies, services, or governance issues separately, leaving limited evidence on how the major components of digital health governance are combined within national policy texts. Second, the studies reviewed above do not provide a unified policy-evaluation framework that fully represents the distinctive requirements of digital health, particularly the relationships among data resources, foundational platforms, standards, digitally enabled services, regulatory governance, data protection, innovation, and implementation support. Third, reproducible cross-policy comparison requires explicit reporting of indicator definitions, coding rules, source selection, and reliability procedures. These gaps limit systematic comparison of what national digital health policies explicitly cover and where structural differences arise.

To address these limitations, this study develops a domain-specific PMC framework based on a source-locked corpus and a fully specified 48-indicator codebook. It addresses three questions: (1) Which dimensions of China’s national digital health policies receive the most and least consistent structural coverage? (2) How do individual policies differ in their combinations of objectives, data, platforms, standards, services, governance, protection, innovation, and supporting mechanisms? (3) Are the principal structural patterns robust to the influence of individual policies and closely related policy families? By combining descriptive text mining, evidence-linked binary coding, inter-coder reliability assessment, and robustness analysis, the study aims to provide a reproducible comparison of policy-text structure without equating textual coverage with implementation effectiveness or policy outcomes.

## 2 Materials and methods

### 2.1 Data sources, eligibility, and source lock

The study considered national-level Chinese policy documents within the search window 2016-01-01–2025-12-01. Policy issuance or signature date, rather than web-posting or archive date, was used as the date convention. A policy was eligible only when digital technology, health data, information platforms, digitally enabled services, digital governance, or artificial intelligence constituted a primary policy object or a substantive dedicated operational component. Incidental mentions of digitalization were insufficient. The unit of analysis was the complete policy text rather than a news report, interpretation page, or issuance cover page.

Forty-two eligible policies were identified. A policy entered the primary analytic corpus only when its title and identifying metadata matched and its complete text could be archived in an auditable canonical form with raw-file and normalized-text SHA-256 hashes. Official central-government and ministry sources were preferred. When direct central access was unavailable, complete official provincial or local mirrors, or stable professional archives with verifiable central provenance, could be used under the predefined source hierarchy. Thirty-nine policies met the source-lock requirements (Table 1). N1, N2, and N21 were retained as eligible, identified reserve policies but excluded from quantitative coding because complete auditable text could not be archived or reliably converted; they were not classified as irrelevant.

**Table 1:** Source-locked national digital health policy corpus (n = 39).

Code	Policy title	Year	Function stratum
P1	Guiding Opinions of the General Office of the State Council on Promoting and Regulating the Application and Development of Health and Medical Big Data	2016	Data/platform/standards
P2	Healthy China 2030 Planning Outline	2016	Strategic/comprehensive
P5	Guiding Opinions of the General Office of the State Council on Advancing the Development of Medical Consortia	2017	Digital service delivery
P6	Special Plan for Health Science and Technology Innovation under the 13th Five-Year Plan	2017	Innovation/AI
P7	Opinions on Accelerating the Popularization and Application of Electronic Health Cards	2018	Data/platform/standards
P8	Opinions of the General Office of the State Council on Promoting the Development of Internet Plus Healthcare	2018	Digital service delivery
P9	Notice on Deepening Internet Plus Healthcare Initiatives for Convenient and Beneficial Services	2018	Digital service delivery
P10	Measures for the Administration of National Health and Medical Big Data Standards, Security, and Services (Trial)	2018	Security/regulation
P11	Measures for the Administration of Internet Diagnosis and Treatment (Trial)	2018	Digital service delivery
P12	Measures for the Administration of Internet Hospitals (Trial)	2018	Digital service delivery
P13	Standards for the Administration of Telemedicine Services (Trial)	2018	Digital service delivery
P14	Graded Evaluation Standards for the Application Level of Electronic Medical Record Systems	2018	Data/platform/standards
P16	Notice on Further Improving the Appointment-Based Diagnosis and Treatment System and Strengthening Smart Hospital Development	2020	Digital service delivery
P17	Opinions on Strengthening the Standardization System for National Health Informatization	2020	Data/platform/standards
P18	Notice on Deepening the Internet Plus Healthcare Five-in-One Service Initiative	2020	Digital service delivery
P19	Graded Evaluation Standards for Smart Hospital Management (Trial)	2021	Data/platform/standards
P20	14th Five-Year Plan for Universal Medical Security	2021	Payment/insurance digitalization
P21	Special Plan for Health Science and Technology Innovation under the 14th Five-Year Plan	2022	Innovation/AI
P22	14th Five-Year Plan for National Health Informatization	2022	Strategic/comprehensive
P23	Standards for Information and Digital Development of Traditional Chinese Medicine Hospitals (2024 Edition)	2024	Data/platform/standards
P24	Implementation Opinions on Promoting and Regulating the Development of Artificial Intelligence Plus Healthcare Applications	2025	Innovation/AI
N3	13th Five-Year Plan for National Population Health Informatization	2017	Strategic/comprehensive
N6	Technical Guidelines for the Application of Hospital Informatization (2017 Edition)	2017	Data/platform/standards
N7	National Standards and Specifications for Hospital Informatization Development (Trial)	2018	Data/platform/standards
N8	Notice on Further Advancing Informatization in Medical Institutions with Electronic Medical Records at the Core	2018	Data/platform/standards
N10	Graded Evaluation Standards for Smart Hospital Services (Trial)	2019	Digital service delivery
N11	National Standards and Specifications for Informatization Development in Primary Healthcare Institutions (Trial)	2019	Data/platform/standards
N12	Guiding Opinions on Improving Pricing and Medical Insurance Payment Policies for Internet Plus Medical Services	2019	Payment/insurance digitalization
N13	Guiding Opinions on Advancing Medical Insurance Payment for Internet Plus Medical Services	2020	Payment/insurance digitalization
N14	National Standards and Specifications for Public Health Informatization Development (Trial)	2020	Data/platform/standards
N15	Detailed Rules for the Regulation of Internet Diagnosis and Treatment (Trial)	2022	Security/regulation
N16	Measures for Cybersecurity Management in Medical and Health Institutions	2022	Security/regulation
N17	Administrative Standards for the Application of Clinical Decision Support Systems in Medical Institutions (Trial)	2023	Innovation/AI
N20	Reference Guidelines for Artificial Intelligence Application Scenarios in the Health Sector	2024	Innovation/AI
N22	13th Five-Year Plan for Traditional Chinese Medicine Informatization Development	2016	Strategic/comprehensive
N23	Guiding Opinions on Accelerating the Reform of Electronic Registration Management for Medical Institutions, Physicians, and Nurses	2017	Data/platform/standards
N25	Guiding Opinions of the National Health Commission on Strengthening Health Statistics	2020	Data/platform/standards
N26	Guiding Opinions of the National Healthcare Security Administration on Strengthening Cybersecurity and Data Protection	2021	Security/regulation
N32	Guiding Opinions on Further Advancing Mutual Recognition of Medical Examination and Test Results	2024	Digital service delivery

The analysis proceeded through source locking, UTF-8 text normalization, paragraph-level tokenization, descriptive text mining, codebook-guided binary coding, PMC aggregation, reliability assessment, and prespecified robustness analyses. Fig 1 documents policy identification, source lock, and formation of the 39-policy analytic corpus.

**Fig 1:**
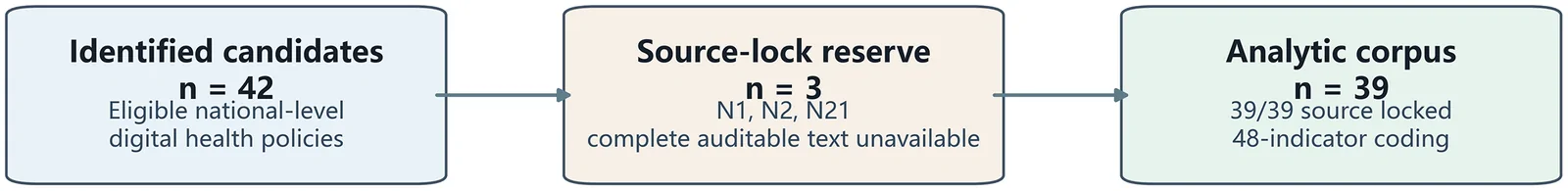
Policy identification, source lock, and analytic corpus flow.

### 2.2 Text preprocessing and semantic network analysis

Canonical policy texts were decoded strictly as UTF-8 or UTF-8 with byte-order mark and processed in Python. The pipeline retained 21,143 substantive paragraphs across the 39 documents. Chinese segmentation used jieba 0.42.1 in accurate mode with HMM disabled, supplemented by a project dictionary and a documented stop-word list. The processed corpus contained 151,273 retained tokens and 7,828 distinct terms. These deterministic steps were checked against the recorded text hashes.

Word frequencies were calculated after removing administrative boilerplate and low-information function words while retaining substantively meaningful governance terms. Document frequency was recorded alongside total frequency to distinguish corpus-wide vocabulary from terms concentrated in a small number of policies. Fig 2 display the 20 highest-frequency terms with document frequency of at least five after four low-information terms were excluded from the display only. The complete machine-readable frequency table is provided in S1 File.

**Fig 2:**
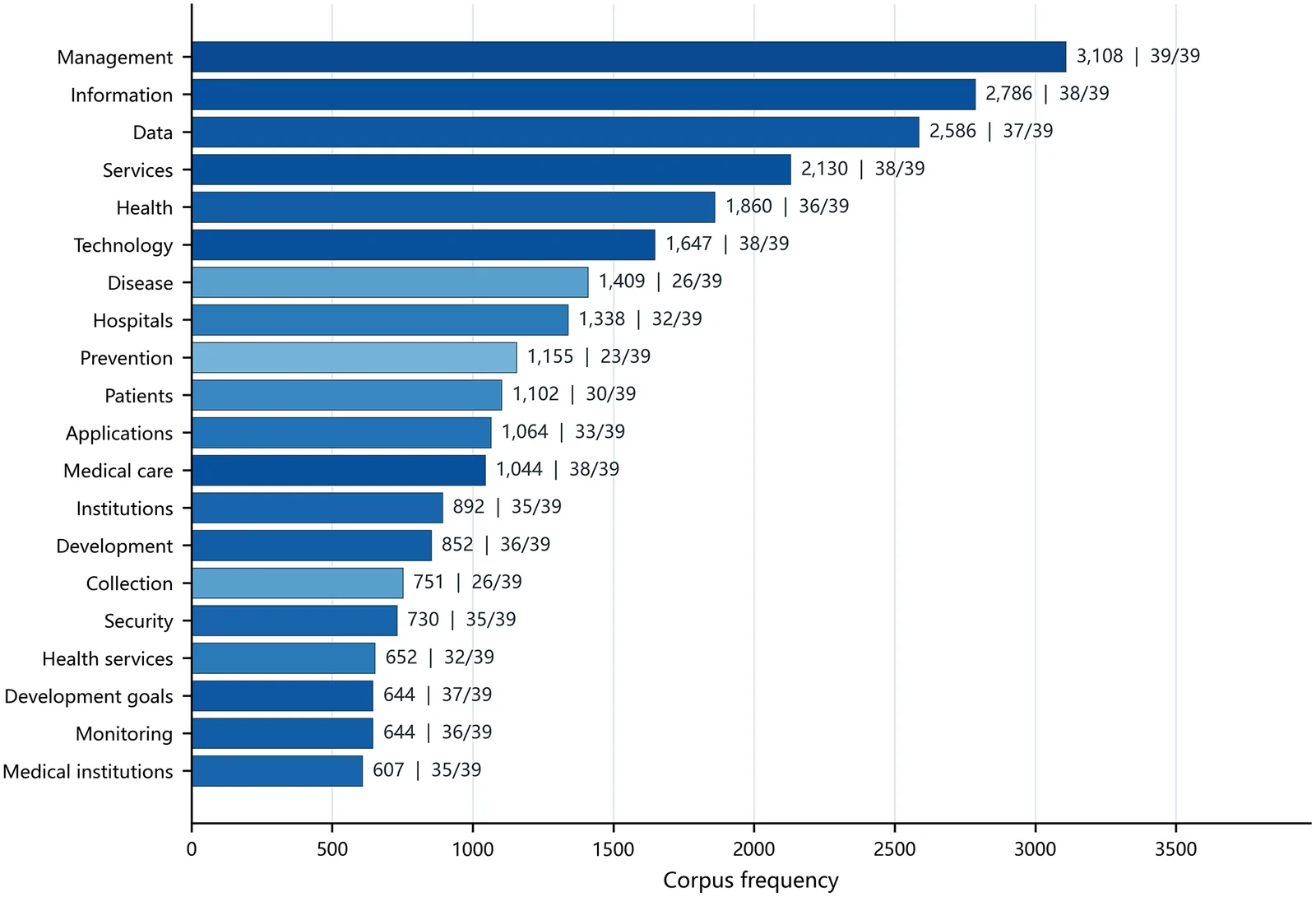
Twenty most frequent displayed terms in the 39-policy corpus. Bars show token frequency; labels show token and policy-document frequencies.

For the semantic network, two terms were linked when they co-occurred in the same retained paragraph. Aggregation over all documents produced 406,137 weighted term-pair edges. Fig 3 displays the 30 highest-frequency nodes after the same four low-information display terms were excluded. Among eligible displayed-node edges, a maximum spanning tree first ensured connectivity and the strongest remaining edges were then added until 60 edges were shown. Node size represents corpus frequency, node color weighted degree, and edge width paragraph-level co-occurrence. The complete machine-readable node and edge tables are provided in S1 File. Text mining was descriptive and did not determine binary PMC scores.

**Fig 3:**
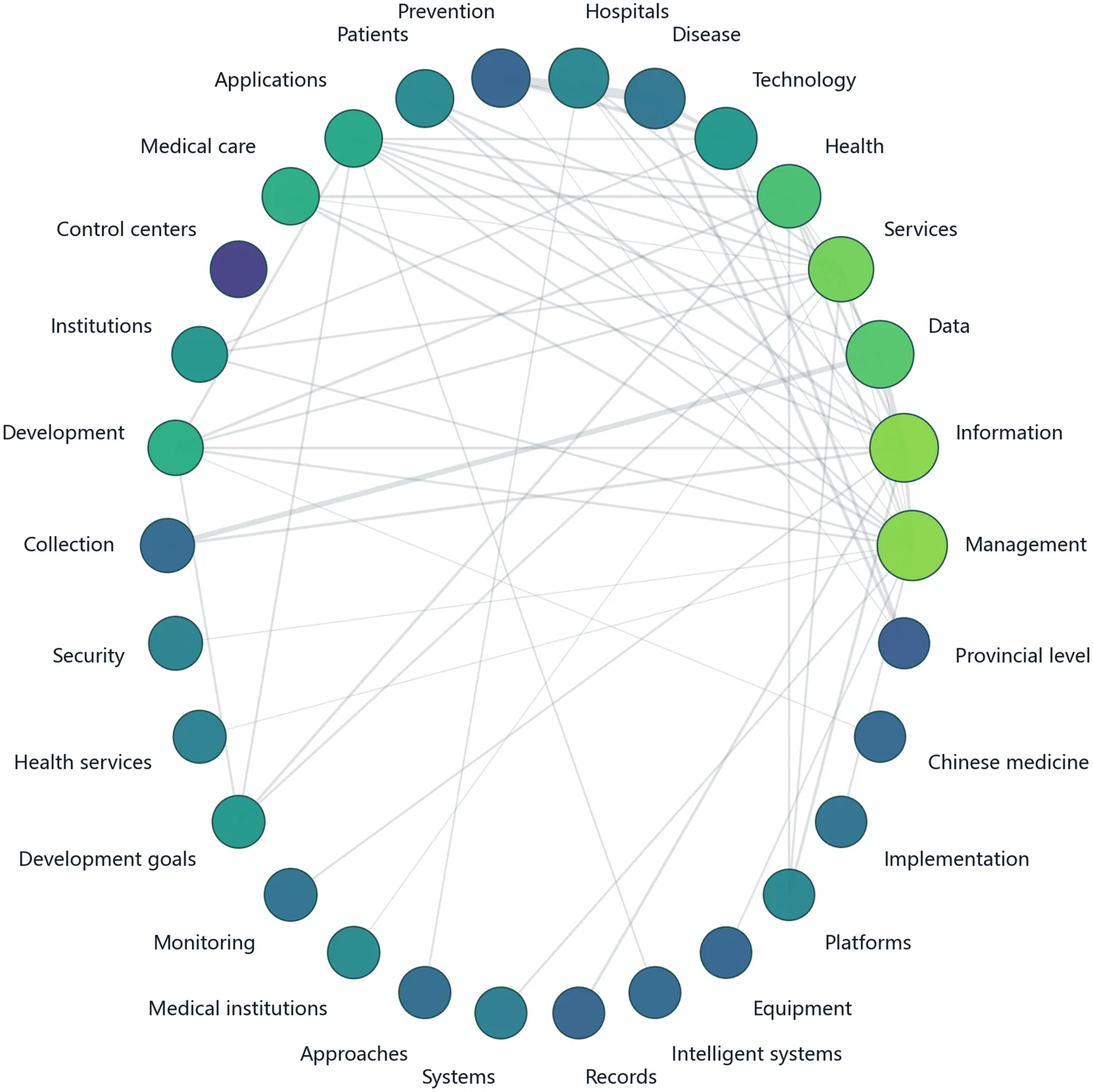
Displayed paragraph-level semantic co-occurrence network. Node size represents corpus frequency, node color represents weighted degree, and edge width represents paragraph-level co-occurrence.

### 2.3 PMC framework and coding procedure

The PMC approach decomposes policy texts into observable elements and compares documents under a common coding framework [30]. Here it was used as a measure of structural coverage: whether specified elements appeared in a policy text. It was not treated as a direct measure of implementation quality, policy effectiveness, or policy outcomes.

The framework was contextualized using PMC-oriented internet-healthcare policy analysis [31], research on digital-health services and rural access [21], evidence on digital-health literacy in China [32], and legal scholarship on Chinese health-data protection [28]. Codebook v1.0 contains nine first-level dimensions and 48 second-level indicators: X1 policy objectives (5), X2 data resources (6), X3 foundational platforms (6), X4 standards and institutional arrangements (5), X5 application and service delivery (6), and X6-X9 regulatory governance, data protection, industrial innovation, and coordination and enabling mechanisms (5 each). Table 2 gives the complete definitions and binary criteria, including separately coded platform maintenance (X3-5) and iterative upgrading/scalability (X3-6).

**Table 2:** Complete 48-indicator PMC codebook and binary coding criteria.

First-level dimension	Level 1	Second-level indicator	Level 2	Binary criterion
X1 Policy objectives	X1	Vision and objectives	X1-1	Policy explicitly states an overall vision, strategic goal, or general objective for digital health.
X1 Policy objectives	X1	Roadmap	X1-2	Policy specifies phased targets, a timeline, milestones, or an explicit implementation roadmap.
X1 Policy objectives	X1	Task specification	X1-3	Policy identifies concrete key tasks, priority actions, programs, or implementation items.
X1 Policy objectives	X1	Scope of coverage	X1-4	Policy explicitly defines target populations, institutions, sectors, or geographical coverage.
X1 Policy objectives	X1	Primary-level inclusiveness	X1-5	Policy explicitly addresses primary-level capacity, underserved/remote groups, regional balance, or inclusion.
X2 Data resources	X2	Data infrastructure	X2-1	Policy proposes building or improving foundational health data resources such as EHR/EMR, registries, databases, or core health datasets.
X2 Data resources	X2	Data catalog	X2-2	Policy specifies data catalogs, master indexes, resource directories, or equivalent data-resource indexing systems.
X2 Data resources	X2	Data sharing	X2-3	Policy proposes cross-departmental, cross-regional, cross-institutional data sharing or exchange mechanisms.
X2 Data resources	X2	Data openness and authorization	X2-4	Policy explicitly addresses data openness, authorized access/use, controlled circulation, or access authorization.
X2 Data resources	X2	Data governance	X2-5	Policy specifies data classification, quality management, lifecycle governance, rights/responsibilities, or other governance rules.
X2 Data resources	X2	Data-enabled applications	X2-6	Policy proposes using data for healthcare management, research, decision support, public health, or service delivery.
X3 Foundational platforms	X3	Platform architecture	X3-1	Policy proposes or emphasizes national/regional health information platforms or other foundational digital-health platform architecture.
X3 Foundational platforms	X3	Interoperability	X3-2	Policy explicitly requires interoperability, system connectivity, cross-system exchange, or standardized interfaces for information exchange.
X3 Foundational platforms	X3	Cloud infrastructure	X3-3	Policy proposes cloud platforms, cloud storage, cloud imaging, or cloud-based infrastructure supporting digital-health functions.
X3 Foundational platforms	X3	Identity authentication	X3-4	Policy includes unified identity authentication, digital certificates/signatures, trusted identity, or equivalent authentication mechanisms.
X3 Foundational platforms	X3	Platform operation and maintenance	X3-5	Policy explicitly provides for platform/system operation, maintenance, technical support, continuity, backup/recovery, or operational service assurance.
X3 Foundational platforms	X3	Iterative upgrading and scalability	X3-6	Policy explicitly provides for upgrading, continuous improvement, iterative expansion, capacity scaling, system evolution, or adaptation to new technologies/business needs.
X4 Standards and institutional arrangements	X4	Technical standards	X4-1	Policy specifies technical, data, interface, coding, security, or other digital-health standards.
X4 Standards and institutional arrangements	X4	Institutional norms	X4-2	Policy establishes institutional arrangements, operating rules, management rules, access rules, or formal procedures.
X4 Standards and institutional arrangements	X4	Standard coordination	X4-3	Policy emphasizes alignment, harmonization, unification, or coordination among standards, systems, codes, or institutional rules.
X4 Standards and institutional arrangements	X4	Standard implementation and application	X4-4	Policy explicitly requires implementation, application, compliance, conformity testing, or operational adoption of relevant standards.
X4 Standards and institutional arrangements	X4	Standard evaluation and revision	X4-5	Policy provides for assessment of standard application, maturity/conformity evaluation, monitoring/feedback, or revision/update/abolition of standards based on evaluation.
X5 Application and service delivery	X5	Internet-based healthcare services	X5-1	Policy proposes internet-based diagnosis, treatment, consultation, remote care, online follow-up, or equivalent healthcare services.
X5 Application and service delivery	X5	Smart hospital development	X5-2	Policy addresses smart-hospital development or substantive digital transformation of healthcare institutions and clinical workflows.
X5 Application and service delivery	X5	Primary and public health services	X5-3	Policy proposes digital support for primary care, family-doctor services, public health, disease surveillance, or community health services.
X5 Application and service delivery	X5	Service accessibility	X5-4	Policy explicitly aims to improve convenience, continuity, reach, affordability, accessibility, or access for underserved groups through digital means.
X5 Application and service delivery	X5	Integrated and tiered service coordination	X5-5	Policy specifies online-offline integration, cross-level referral, medical-consortium coordination, cross-institution service coordination, or integrated delivery across levels.
X5 Application and service delivery	X5	Closed-loop and continuous service process	X5-6	Policy specifies an end-to-end or longitudinal service process, such as pre-diagnosis–diagnosis–post-diagnosis, prevention–treatment–rehabilitation–health management, follow-up tracking, feedback, or closed-loop management.
X6 Regulatory governance	X6	Regulatory mechanisms	X6-1	Policy specifies regulatory frameworks or formal regulatory mechanisms for digital health.
X6 Regulatory governance	X6	Supervision mechanisms	X6-2	Policy specifies supervision, inspection, oversight, accountability, reporting, or enforcement mechanisms.
X6 Regulatory governance	X6	Multi-actor governance	X6-3	Policy establishes multi-stakeholder, cross-sector, or cross-department governance arrangements.
X6 Regulatory governance	X6	Platform governance	X6-4	Policy explicitly governs digital-health platforms, internet hospitals, data platforms, third-party platforms, or platform operators.
X6 Regulatory governance	X6	Compliance and risk control	X6-5	Policy specifies compliance enforcement, admission/exit rules, credit regulation, risk mitigation, incident handling, or equivalent risk controls.
X7 Data protection	X7	Security responsibilities	X7-1	Policy clearly assigns cybersecurity/data-security responsibilities to institutions, departments, operators, or responsible persons.
X7 Data protection	X7	Security assessment	X7-2	Policy specifies security review, risk assessment, graded protection, security testing, or equivalent assessment mechanisms.
X7 Data protection	X7	Monitoring and early warning	X7-3	Policy specifies cybersecurity/data-security monitoring, alerts, incident reporting, or emergency response mechanisms.
X7 Data protection	X7	Data classification and personal information protection	X7-4	Policy includes data classification/tiering and/or explicit personal information/privacy protection requirements.
X7 Data protection	X7	Critical infrastructure protection	X7-5	Policy explicitly addresses critical information infrastructure, core systems, infrastructure protection, or situational awareness at a comparable level.
X8 Industrial innovation	X8	Key technologies	X8-1	Policy explicitly supports R&D or development of key digital-health technologies such as AI, big data, robotics, digital therapeutics, or related core technologies.
X8 Industrial innovation	X8	Intelligent devices	X8-2	Policy promotes R&D, deployment, or scaling of wearable devices, monitoring equipment, medical robots, intelligent medical devices, or comparable devices.
X8 Industrial innovation	X8	Industrial ecosystem	X8-3	Policy explicitly promotes digital-health industry cultivation, new business models, enterprise development, market ecosystems, or industrial-chain development.
X8 Industrial innovation	X8	Pilot demonstration and translation	X8-4	Policy specifies pilot projects, demonstrations, technology transfer/translation, scaling-up, or replication of innovation.
X8 Industrial innovation	X8	Industry–academia–medicine collaboration	X8-5	Policy explicitly encourages collaborative innovation among enterprises and healthcare/research/academic institutions (at least two substantive actor categories).
X9 Coordination and enabling mechanisms	X9	Organizational coordination	X9-1	Policy establishes leadership arrangements, interdepartmental coordination, task division, or implementation coordination.
X9 Coordination and enabling mechanisms	X9	Financial support	X9-2	Policy specifies funding sources, fiscal support, investment guarantees, reimbursement/payment support, or diversified investment mechanisms for implementation.
X9 Coordination and enabling mechanisms	X9	Talent development	X9-3	Policy includes workforce training, talent development, professional capacity building, or dedicated staffing arrangements for digital health.
X9 Coordination and enabling mechanisms	X9	Monitoring and evaluation	X9-4	Policy specifies implementation monitoring, performance evaluation, assessment indicators, supervision of implementation, or structured feedback.
X9 Coordination and enabling mechanisms	X9	Pilot diffusion	X9-5	Policy provides for pilot implementation plus experience summarization, replication, diffusion, or scale-up mechanisms.

Each indicator received 1 only when the policy text contained explicit, indicator-matched content; absence, purely aspirational language, or a non-specific statement received 0. Coding was led by the primary researcher, who individually reviewed and finalized all 1,872 policy-by-indicator decisions using the predefined 48-indicator codebook and original source texts. Ambiguous and boundary cases received additional scrutiny during calibration. AI-assisted tools were used only as auxiliary aids for source-passage retrieval, text location, evidence organization, and consistency checking; they did not determine final indicator scores. A second human researcher independently coded a prespecified stratified 11-policy subset for inter-coder reliability assessment.

### 2.4 PMC aggregation and structural-coverage categories

For each policy, the binary scores were averaged within each first-level dimension so that dimensions with different numbers of second-level indicators contributed equally. The nine dimension scores were then summed to produce a PMC value ranging from 0 to 9, as follows: Xi denotes the score for dimension i, xij is the binary score for indicator j within that dimension, and ni is the number of indicators assigned to dimension i.

Binary Scoring


$$\begin{array}{c} X_{ij}=\left\{ \begin{array}{c} \text{1},\text{If a policy document explicitly covers the }j-th\text{ second}-\text{level}\\ \;\text{element under the }i-th\text{ first}-\text{level indicator},\\ \text{0},\text{Otherwise},\; \end{array}\right. \end{array}$$

Level-1 Aggregation


$$\begin{array}{c} X_{i}=\frac{\text{1}}{n_{i}}\sum_{j=\text{1}}^{n_{i}}X_{ij}\;(i=\text{1},\text{2},\text{3}\ldots .,\text{9}) \end{array}$$

PMC Index


$$\begin{array}{c} PMC=\sum_{i=\text{1}}^{\text{9}}X_{i} \end{array}$$

For descriptive presentation, PMC scores were grouped using study-defined cut points informed by PMC classification practice [33]: poor (PMC < 4.50), acceptable (4.50 <= PMC < 6.50), good (6.50 <= PMC < 8.00), and perfect (8.00 <= PMC <=9.00). Table 3 summarizes these categories. These labels refer only to structural coverage under this codebook. They do not establish implementation quality or effectiveness.

**Table 3:** Descriptive categories for PMC structural coverage.

PMC index interval	<4.50	4.50-6.49	6.50-7.99	8.00-9.00
Structural coverage category	Poor	Acceptable	Good	Perfect

### 2.5 Reliability assessment

A second human coder independently evaluated a prespecified stratified subset of 11 policies without access to the primary researcher’s coding results. The subset comprised 528 paired policy-by-indicator decisions (11 x 48). Agreement was 96.78%, with Cohen’s kappa = 0.914; 17 disagreements were reviewed against the original policy texts and the predefined codebook. Final adjudicated scores were determined by the primary researcher after re-examination of the source texts and coding criteria. The second coder did not code the remaining 28 policies, so the reliability estimate applies to the prespecified subset rather than the full corpus.

### 2.6 Statistical analysis and robustness checks

Descriptive statistics were calculated for policy-level PMC values and dimension scores. To avoid part-whole circularity, the association for each dimension Xi was assessed against the PMC total excluding Xi, using Spearman’s rank correlation. The 95% confidence intervals were percentile-bootstrap intervals based on 5,000 policy-level resamples. Benjamini-Hochberg false-discovery-rate correction was applied across the nine dimension-level tests. Main-text robustness analyses comprised leave-one-policy-out recomputation and collapse of the closely related P11/P12/P13 policy family. For the policy-family sensitivity analysis, P11, P12, and P13 were replaced by a single union-of-coverage profile in which each binary indicator was scored 1 if any of the three policies scored 1 for that indicator (indicator-wise maximum). A supplementary hypothetical bit-flip stress test at 3%, 5%, and 10% was retained as a sensitivity analysis but was not treated as primary robustness evidence. Analyses were implemented in Python from the locked coding matrix.

## 3 Results

### 3.1 Text-mining results

Across the 39-policy corpus, the most frequent retained terms were management (3,108 occurrences; 39 documents), information (2,786; 38), data (2,586; 37), service (2,130; 38), and health (1,860; 36). Technology, disease, hospital, prevention, patient, application, and medical were also prominent (Table 4; Fig 2). The combination of high term frequency and broad document frequency indicates that management, information, data, and service were corpus-wide themes rather than artifacts of a few long documents.

**Table 4:** Twenty most frequent retained terms in the 39-policy corpus.

Rank	Term	Frequency	Rank	Term	Frequency
1	Management	3108	11	Applications	1064
2	Information	2786	12	Medical care	1044
3	Data	2586	13	Institutions	892
4	Services	2130	14	Development	852
5	Health	1860	15	Collection	751
6	Technology	1647	16	Security	730
7	Disease	1409	17	Health services	652
8	Hospitals	1338	18	Development goals	644
9	Prevention	1155	19	Monitoring	644
10	Patients	1102	20	Medical institutions	607

The paragraph-level semantic network contained 406,137 weighted edges before display filtering (Fig 3). Management, information, data, service, technology, medical, and health occupied central positions, linking institutional, technical, and service-delivery vocabulary. The network is descriptive evidence of recurring thematic relationships; it does not by itself imply causal or implementation relationships.

### 3.2 Policy-level PMC structural coverage

The 39 policy PMC scores ranged from 3.467 to 9.000, with a mean of 6.654 (SD 1.456) and a median of 6.500 (Table 5). Nine policies were classified as perfect, 11 as good, 16 as acceptable, and three as poor in structural coverage. The distribution therefore spans all four descriptive categories and shows substantial heterogeneity in the number and combination of covered elements.

**Table 5:** Dimension scores, PMC values, ranks, and structural-coverage categories for 39 policies.

Code	X1	X2	X3	X4	X5	X6	X7	X8	X9	PMC	Rank	Category
N3	1.000	1.000	1.000	1.000	1.000	1.000	1.000	1.000	1.000	9.000	1	Perfect
P22	1.000	1.000	0.833	1.000	1.000	1.000	1.000	1.000	1.000	8.833	2	Perfect
P1	1.000	1.000	1.000	1.000	1.000	1.000	0.800	1.000	1.000	8.800	3	Perfect
N22	1.000	1.000	0.833	1.000	1.000	1.000	0.800	1.000	1.000	8.633	4	Perfect
P2	0.600	1.000	1.000	1.000	0.833	1.000	0.600	1.000	1.000	8.033	5	Perfect
N6	0.800	1.000	1.000	1.000	1.000	1.000	0.800	0.600	0.800	8.000	6	Perfect
N7	0.800	1.000	1.000	1.000	1.000	1.000	0.800	0.600	0.800	8.000	6	Perfect
N14	0.800	1.000	1.000	1.000	1.000	1.000	0.800	0.600	0.800	8.000	6	Perfect
P8	0.600	1.000	1.000	1.000	1.000	1.000	0.600	1.000	0.800	8.000	6	Perfect
P17	1.000	1.000	0.500	1.000	0.833	1.000	0.800	1.000	0.800	7.933	10	Good
P23	0.800	1.000	0.833	1.000	0.833	1.000	1.000	0.600	0.800	7.867	11	Good
P18	0.600	1.000	0.833	1.000	1.000	1.000	0.400	1.000	1.000	7.833	12	Good
N20	1.000	0.833	1.000	1.000	1.000	0.800	0.600	1.000	0.600	7.833	12	Good
P24	0.600	0.833	0.833	1.000	0.833	1.000	0.800	1.000	0.800	7.700	14	Good
N11	0.800	1.000	0.833	1.000	1.000	1.000	0.600	0.600	0.800	7.633	15	Good
P20	1.000	0.833	0.667	1.000	0.833	1.000	0.400	0.800	1.000	7.533	16	Good
P7	0.600	1.000	0.667	1.000	1.000	0.800	0.200	1.000	0.800	7.067	17	Good
N25	1.000	1.000	0.333	0.800	0.667	1.000	0.600	0.600	0.800	6.800	18	Good
N23	0.600	0.833	0.833	0.800	0.667	1.000	0.800	0.200	1.000	6.733	19	Good
P11	0.400	0.833	0.833	0.800	0.833	1.000	0.600	0.600	0.600	6.500	20	Good
N16	0.800	0.500	0.667	1.000	0.500	0.800	1.000	0.400	0.800	6.467	21	Acceptable
P21	0.800	0.500	0.500	1.000	0.833	0.800	0.200	1.000	0.800	6.433	22	Acceptable
P9	0.600	0.667	0.833	0.800	1.000	0.600	0.200	0.800	0.800	6.300	23	Acceptable
N26	0.400	1.000	0.500	0.800	0.333	1.000	1.000	0.200	1.000	6.233	24	Acceptable
P5	1.000	0.667	0.500	0.400	0.833	0.600	0.200	0.800	1.000	6.000	25	Acceptable
P16	0.400	0.667	0.500	0.800	1.000	1.000	0.200	0.800	0.600	5.967	26	Acceptable
P19	0.400	1.000	0.500	1.000	0.667	0.800	0.600	0.200	0.800	5.967	26	Acceptable
N8	0.600	0.833	0.833	0.800	0.833	0.600	0.800	0.200	0.400	5.900	28	Acceptable
P10	0.200	1.000	0.500	1.000	0.333	1.000	1.000	0.200	0.600	5.833	29	Acceptable
P6	1.000	0.333	0.333	1.000	0.500	0.600	0.000	1.000	1.000	5.767	30	Acceptable
N10	0.800	1.000	0.333	0.800	1.000	0.800	0.400	0.400	0.200	5.733	31	Acceptable
N32	0.800	0.833	0.500	0.800	0.500	0.600	0.200	0.400	0.800	5.433	32	Acceptable
P12	0.400	0.500	0.667	0.200	0.667	1.000	0.600	0.400	0.400	4.833	33	Acceptable
N13	0.200	0.667	0.500	0.400	0.667	1.000	0.000	0.600	0.800	4.833	33	Acceptable
N15	0.200	0.667	0.500	0.400	0.667	1.000	0.600	0.200	0.600	4.833	33	Acceptable
N12	0.600	0.333	0.000	0.600	0.833	1.000	0.000	0.400	0.800	4.567	36	Acceptable
P13	0.400	0.167	0.667	0.800	0.500	0.800	0.200	0.400	0.400	4.333	37	Poor
P14	0.600	0.667	0.333	0.800	0.667	0.600	0.000	0.000	0.200	3.867	38	Poor
N17	0.200	0.667	0.667	0.400	0.333	0.400	0.400	0.200	0.200	3.467	39	Poor
Mean	0.677	0.816	0.684	0.851	0.795	0.887	0.554	0.636	0.754	6.654		Good

The 39 x 9 dimension heatmap (Fig 4) shows that high and low values were distributed across multiple policy families rather than confined to a single period or issuer type. The sorted PMC plot (Fig 5) further shows a continuous score distribution, including policies near category thresholds. Together, the two views support comparison of both within-policy profiles and between-policy totals.

**Fig 4:**
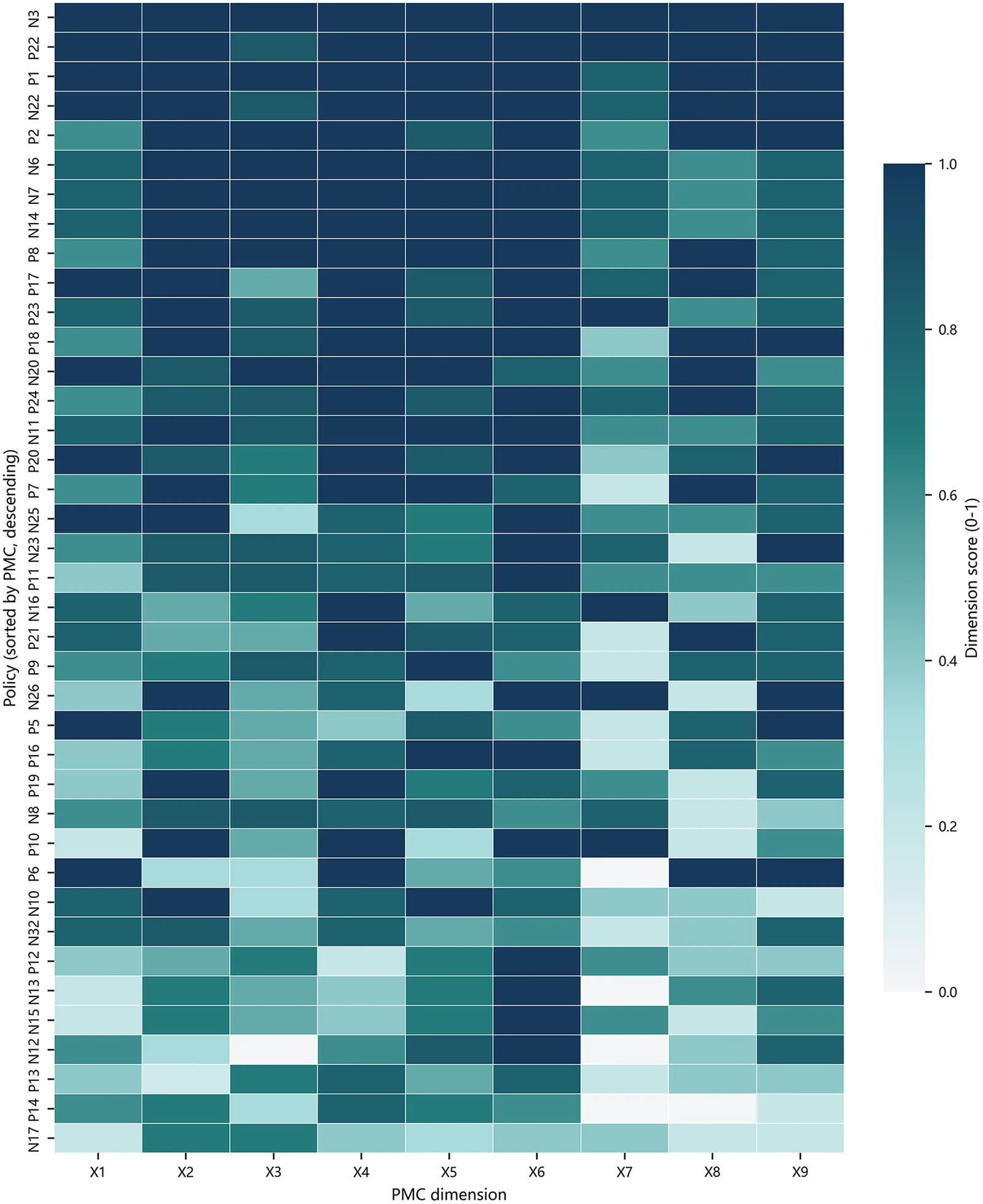
Structural-coverage heatmap for 39 policies across nine dimensions.

**Fig 5:**
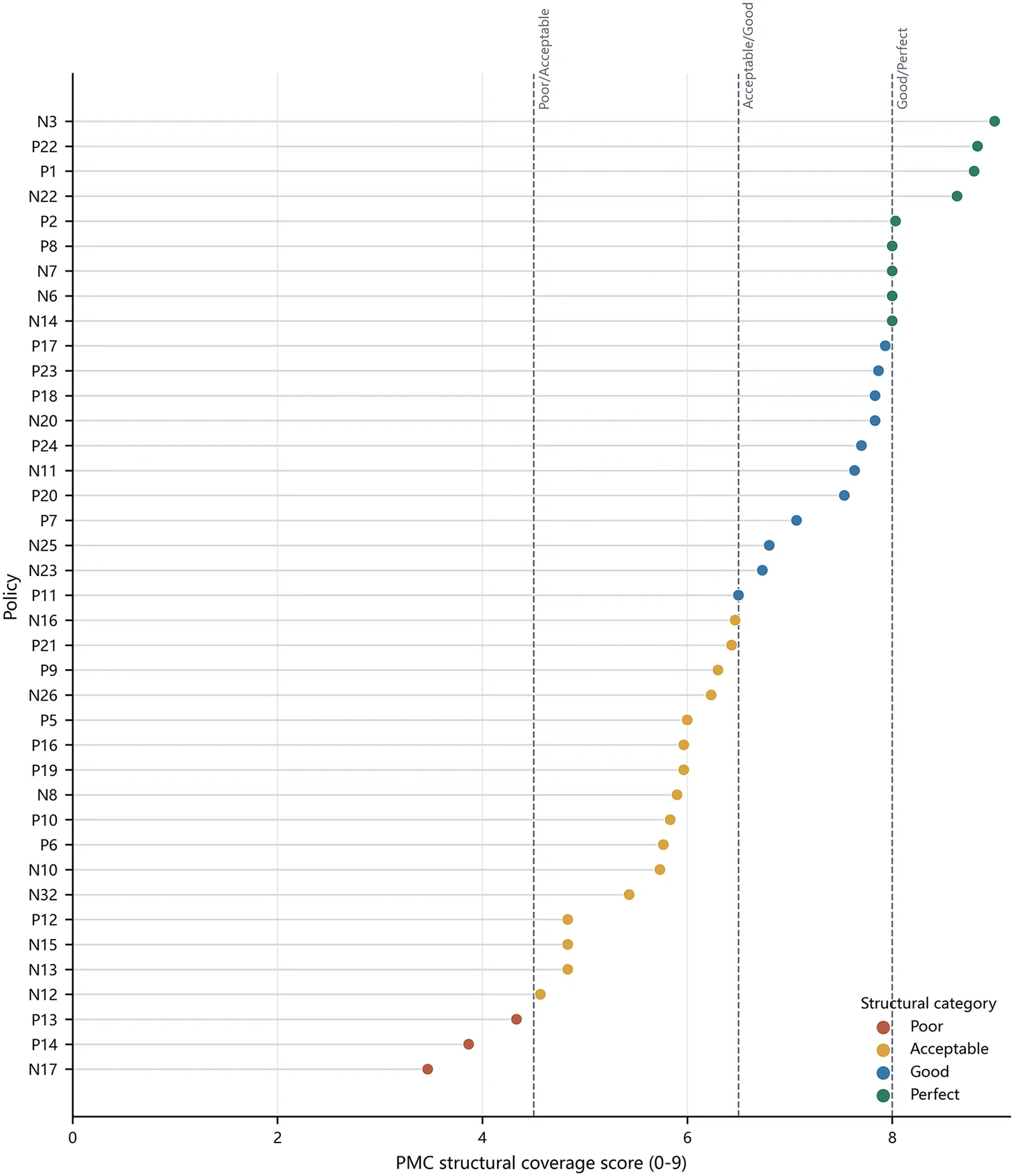
Sorted PMC structural-coverage scores for 39 policies.

Comprehensive and strategic policies generally covered more dimensions, whereas specialized documents often displayed deliberately concentrated profiles. A lower PMC value for a specialized policy therefore indicates narrower textual scope under the common 48-indicator framework; it should not be interpreted automatically as inferior policy design or weak implementation.

### 3.3 Dimension-level structural coverage

Mean dimension scores ranged from 0.554 to 0.887 (Fig 6). Regulatory governance (X6, 0.887), standards and institutional arrangements (X4, 0.851), and data resources (X2, 0.816) had the highest mean coverage. They were followed by application and service delivery (X5, 0.795), coordination and enabling mechanisms (X9, 0.754), foundational platforms (X3, 0.684), policy objectives (X1, 0.677), industrial innovation (X8, 0.636), and data protection (X7, 0.554).

**Fig 6:**
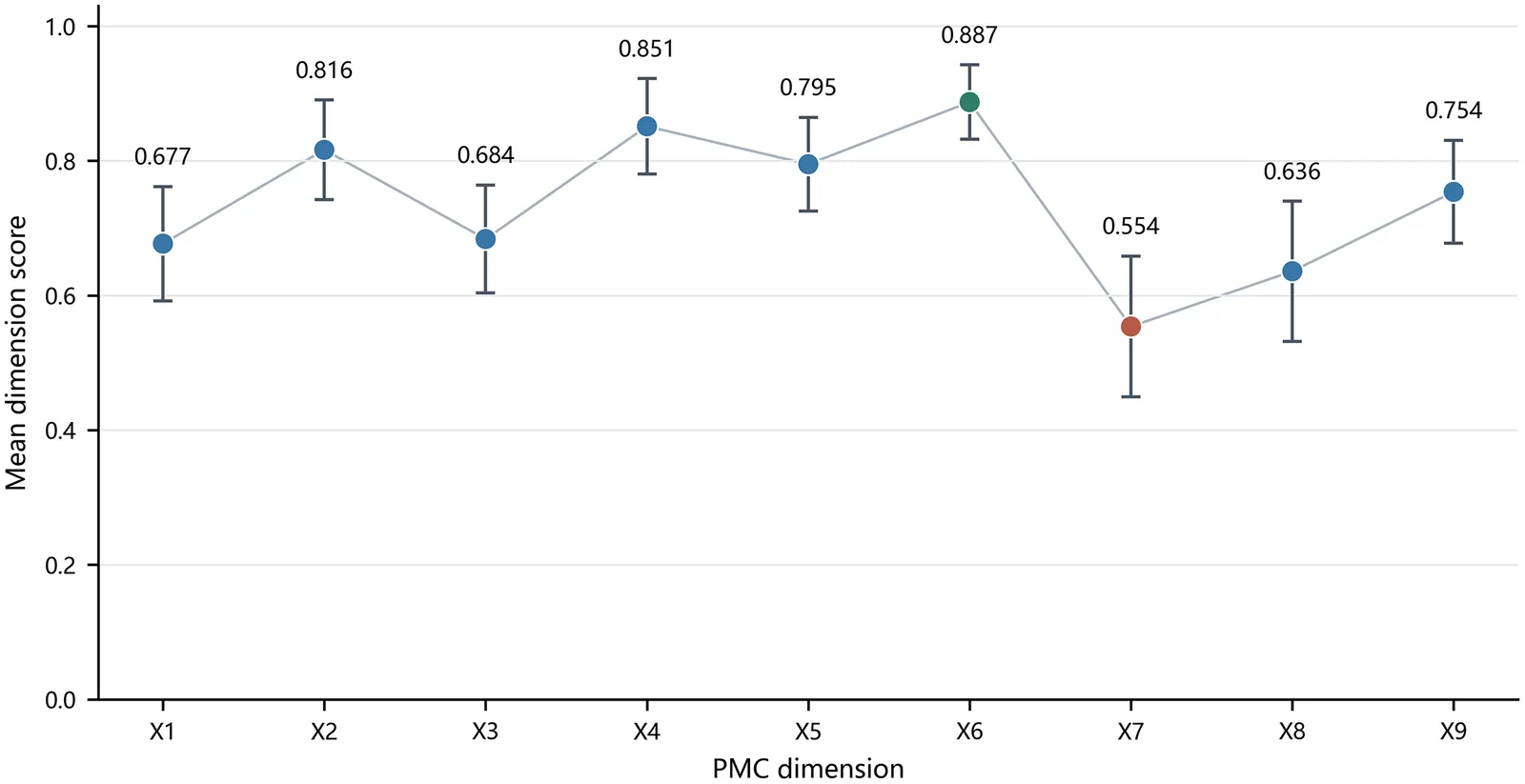
Mean structural coverage and dispersion across nine dimensions. Points show means across 39 policies; error bars are two-sided 95% t confidence intervals.

Variability was greatest for X7 (SD 0.323) and X8 (SD 0.321), indicating that security-related detail and innovation-oriented provisions were unevenly covered across policies. X6 had both the highest mean and the lowest standard deviation (0.170), suggesting broad and comparatively consistent coverage of regulatory arrangements in the sampled texts.

To clarify the components underlying the aggregate PMC profile, the following subsections examine each of the nine dimensions separately, focusing on both overall coverage and the secondary indicators that generated cross-policy variation.

### 3.4 Policy objectives (X1)

Policy objectives had a mean structural-coverage score of 0.677 (SD = 0.262; median = 0.600; range = 0.200–1.000), placing X1 in the middle-to-lower part of the nine-dimension profile. Overall visions, strategic objectives, and concrete task specifications were commonly represented, indicating that most documents clearly articulated what they intended to achieve or undertake. Greater variation emerged in the specification of implementation roadmaps, substantive coverage boundaries, and primary-level or inclusion-oriented objectives. Some policies defined phased tasks, target institutions, populations, or underserved groups in considerable detail, whereas others remained focused on broad strategic goals. The X1 pattern therefore reflects a distinction between stating policy direction and translating that direction into bounded objectives, implementation sequences, and clearly specified target groups.

### 3.5 Data resources (X2)

Data resources showed relatively high structural coverage (mean = 0.816; SD = 0.229; median = 0.833; range = 0.167–1.000). The data-enabled applications indicator (X2-6) was present in all 39 policies, showing that the use of data for healthcare, public health, management, research, or decision support has become a common textual component of the corpus. Nevertheless, substantial differences remained in how policies addressed the underlying data-governance architecture. Data catalogs, authorized access and circulation, cross-institutional sharing, data quality, classification, and lifecycle governance were not combined uniformly across documents. Thus, although the practical use of data is broadly recognized, the institutional and organizational arrangements needed to structure data resources vary considerably across policy functions.

### 3.6 Foundational platforms (X3)

Foundational platforms had a mean score of 0.684 (SD = 0.247; median = 0.667; range = 0–1.000). Platform architecture and interoperability were comparatively prominent components of this dimension, reflecting sustained policy attention to health-information platforms, system connectivity, and standardized information exchange. By contrast, cloud infrastructure and unified identity or authentication mechanisms were less consistently embedded across policy texts. Operation and maintenance (X3-5) and iterative upgrading and scalability (X3-6) were intentionally retained as separate indicators because they represent different stages of platform governance: the former concerns continuity and reliable operation, whereas the latter captures the capacity for technological or functional evolution. Variation across these indicators shows that platform construction alone does not necessarily imply equally explicit provisions for long-term operation, upgrading, or expansion.

### 3.7 Standards and institutional arrangements (X4)

Standards and institutional arrangements were among the most extensively covered dimensions (mean = 0.851; SD = 0.219; median = 1.000; range = 0.200–1.000). Institutional norms (X4-2) were identified in all 39 policies, indicating that formal rules, management arrangements, access requirements, or operating procedures are widely embedded within the policy corpus. However, the broader standards lifecycle was less uniform. Policies varied in whether they specified technical standards, coordinated different standards or codes, required standards to be implemented in practice, or established mechanisms for evaluation, feedback, and revision. Institutional rules were therefore more common than explicit provisions for implementing, evaluating, and updating standards.

### 3.8 Application and service delivery (X5)

Application and service delivery recorded a relatively high mean coverage of 0.795 (SD = 0.214; median = 0.833; range = 0.333–1.000). Policies commonly addressed internet-based healthcare, digitally enabled institutional services, primary or public-health applications, and improvements in service accessibility. Greater differentiation appeared in provisions that required coordination across institutions or service levels and in those describing longitudinal or closed-loop service processes. In particular, integrated online-offline delivery, cross-level referral, inter-institutional coordination, follow-up, and end-to-end service management were not specified with the same consistency as general service provision. The results suggest that digital service availability is a well-established component of national policy texts, while the organizational integration of services across institutions, levels, and stages of care remains more unevenly articulated.

### 3.9 Regulatory governance (X6)

Regulatory governance was the highest-scoring dimension in the framework (mean = 0.887; SD = 0.170; median = 1.000; range = 0.400–1.000) and also showed the lowest dispersion among the nine dimensions. Most policies contained explicit regulatory mechanisms, supervisory arrangements, allocation of governance responsibilities, platform-governance requirements, or provisions for compliance and risk control. The combination of a high mean and relatively limited variation indicates that governance arrangements are a broadly shared structural feature across otherwise heterogeneous policy types. At the same time, the observed range shows that specialized documents do not necessarily cover every regulatory component. The X6 result therefore reflects widespread textual institutionalization of governance mechanisms rather than evidence about the actual strength of enforcement or regulatory performance.

### 3.10 Data protection (X7)

Data protection had the lowest mean structural coverage of all nine dimensions (mean = 0.554; SD = 0.323; median = 0.600; range = 0–1.000) and one of the largest levels of cross-policy dispersion. Provisions concerning cybersecurity and data-security responsibilities, security assessment, monitoring and early warning, personal-information protection, data classification, and critical-infrastructure protection were distributed unevenly across the corpus. Some documents addressed several of these safeguards simultaneously, whereas policies whose principal purposes lay elsewhere often covered only part of the protection framework. This pattern should not be interpreted as evidence of weak cybersecurity performance in practice. Rather, it indicates that explicit data-protection requirements are not equally incorporated into all types of digital health policy texts.

### 3.11 Industrial innovation (X8)

Industrial innovation was the second-lowest dimension (mean = 0.636; SD = 0.321; median = 0.600; range = 0–1.000) and, like data protection, displayed substantial heterogeneity across policies. References to key digital-health technologies and pilot or demonstration activities appeared more frequently than comprehensive combinations of intelligent devices, industrial-ecosystem development, technology translation, and industry–academia–medicine collaboration. This distribution reflects the selective role of innovation provisions within the corpus: they are central to policies focused on science, technology, or artificial intelligence, but less integral to documents concerned primarily with service regulation, standards, or administrative coordination. Accordingly, the relatively low X8 mean represents uneven textual coverage across policy functions rather than limited innovation activity in the digital health sector itself.

### 3.12 Coordination and enabling mechanisms (X9)

Coordination and enabling mechanisms had a mean score of 0.754 (SD = 0.236; median = 0.800; range = 0.200–1.000). Organizational leadership, interdepartmental coordination, and allocation of implementation responsibilities were common components of the policy corpus. By comparison, financial support, dedicated digital-health workforce development, implementation monitoring, and mechanisms for diffusing or scaling pilot experience were not always combined within the same document. Organizational coordination was more consistently specified than financial, workforce, monitoring, and pilot-diffusion support. These supporting functions were often distributed across different policy instruments rather than concentrated within a single document.

Taken together, the dimension-specific results reveal a policy structure in which governance, standards, data resources, and service delivery are comparatively well represented, while protection- and innovation-related provisions are more uneven. The next analysis examines whether coverage in each dimension is systematically associated with the remaining PMC structure and whether the overall pattern is robust to alternative corpus specifications.

### 3.13 Dimension-level association and robustness analyses

All nine dimensions were positively associated with the PMC total excluding that dimension, eliminating the mechanical part-whole contribution present when Xi is correlated with the total containing Xi. Spearman rho ranged from 0.412 for X7 to 0.709 for X4. Bootstrap 95% confidence intervals were positive for every dimension, and all Benjamini-Hochberg adjusted q-values were below 0.01. These associations describe co-occurrence of structural coverage across dimensions rather than predictive accuracy.

Leave-one-policy-out recomputation produced mean PMC values from 6.592 to 6.738; the maximum absolute deviation from the full-corpus mean was 0.084 when N17 was omitted. X6 remained the highest and X7 the lowest dimension in every leave-one-out run (Table 6). Collapsing closely related policy families reduced the analytic set to 37 policies and increased the mean PMC by 0.112 (1.68%); the broad ordering was retained, with only X1 and X3 exchanging adjacent positions (Table 7). Table 8 presents the dimension associations.

**Table 6:** Leave-one-policy-out robustness summary.

Metric	Result	Interpretation
Full-corpus mean PMC	6.653846	Baseline, n = 39
Leave-one-out mean range	6.592105-6.737719	39 omission runs
Maximum absolute deviation	0.083873 (omit N17)	No single policy dominates the mean
Dimension extremes	X6 highest; X7 lowest	Unchanged in all 39 runs

**Table 7:** P11/P12/P13 policy-family collapse robustness summary.

Metric	Primary corpus (n = 39)	Family-collapsed (n = 37)	Interpretation
Analytic units	39	37	P11/P12/P13 replaced by one indicator-wise union profile
Mean PMC	6.653846	6.765766	+0.111920 (+1.682%)
Median PMC	6.500000	6.733333	+0.233333
Highest / lowest dimension	X6 / X7	X6 / X7	Unchanged
Category distribution	9 / 11 / 16 / 3	9 / 11 / 15 / 2	Perfect / Good / Acceptable / Poor
Dimension order	X6 > X4 > X2 > X5 > X9 > X3 > X1 > X8 > X7	X6 > X4 > X2 > X5 > X9 > X1 > X3 > X8 > X7	Only adjacent X1/X3 order changed

**Table 8:** Associations between each dimension and PMC excluding that dimension.

Dimension	N	Spearman rho	p	95% CI low	95% CI high	BH q	Outcome
X1	39	0.504	0.001082	0.210	0.714	0.001623	PMC excluding dimension
X2	39	0.609	0.000038	0.369	0.772	0.000115	PMC excluding dimension
X3	39	0.645	0.000009	0.414	0.790	0.000042	PMC excluding dimension
X4	39	0.709	<0.000001	0.511	0.826	0.000004	PMC excluding dimension
X5	39	0.575	0.000128	0.329	0.754	0.000288	PMC excluding dimension
X6	39	0.436	0.005575	0.140	0.674	0.006271	PMC excluding dimension
X7	39	0.412	0.009126	0.111	0.663	0.009126	PMC excluding dimension
X8	39	0.546	0.000326	0.294	0.729	0.000586	PMC excluding dimension
X9	39	0.468	0.002668	0.154	0.704	0.003431	PMC excluding dimension

Taken together, the leave-one-policy-out and family-collapse analyses indicate that the aggregate dimension pattern is not driven by a single document or the closely related P11/P12/P13 family. Policies close to fixed thresholds should still be interpreted using their continuous scores alongside category labels.

The dimension-level association analysis and robustness checks support internal consistency of the structural-coverage results. They do not validate the PMC score against external implementation or health-outcome criteria, which were outside the scope of this text-based study.

## 4 Discussion

### 4.1 Interpretation

The 39-policy corpus shows an uneven structural profile across the nine dimensions. Regulatory governance (X6), standards and institutional arrangements (X4), and data resources (X2) showed the highest average coverage, whereas data protection (X7) and industrial innovation (X8) showed both lower means and comparatively high dispersion. This pattern suggests that the policy system has developed a relatively extensive institutional and organizational foundation for digital health, while the incorporation of protection- and innovation-related elements remains more dependent on the specific function of individual policy documents.

The strong coverage of X6 and X4 is particularly notable. Regulatory governance recorded not only the highest mean score but also the lowest cross-policy dispersion, while institutional norms were present across the entire corpus. These findings indicate that formal governance arrangements—such as responsibilities, supervision, operating rules, platform regulation, and compliance requirements—constitute a common textual foundation across multiple types of digital health policy. This interpretation is consistent with broader research emphasizing the importance of institutional coordination and governance arrangements in complex health-policy systems [34, 35]. At the same time, the present analysis concerns the presence of such provisions in policy texts and does not establish how effectively they are implemented.

Data resources also showed extensive coverage, with data-enabled applications appearing in every policy in the analytic corpus. However, the secondary indicators reveal an important distinction between using data and governing data. Policies frequently specify how data can support healthcare delivery, management, public health, or decision-making, whereas arrangements concerning catalogs, authorization, sharing, classification, and lifecycle governance are more uneven. This difference helps explain why data resources rank highly overall without being structurally uniform. It also reinforces the value of evaluating individual components rather than treating data governance as a single undifferentiated policy feature.

In contrast, the lower mean and high dispersion of X7 indicate that explicit data-protection provisions are distributed selectively across the policy system. Research on health-data governance and privacy similarly emphasizes the multidimensional nature of protection requirements and privacy safeguards [28, 36]. The present results do not imply that policies with lower X7 scores are insecure in practice; rather, they show that such safeguards are not always articulated within the same policy document. Some of these obligations may be concentrated in dedicated cybersecurity, data-protection, or health-information instruments rather than repeated throughout every specialized service or technology policy.

A similar logic applies to industrial innovation. The relatively low mean and high dispersion of X8 reflect the uneven distribution of R&D, intelligent-device, industrial-ecosystem, pilot-translation, and collaborative-innovation provisions across policy types. Innovation-oriented documents naturally contain more of these elements, whereas regulatory or service-specific instruments may have little reason to reproduce them. Consequently, differences in PMC profiles should be interpreted in relation to policy function. Comprehensive strategic documents have more opportunities to cover multiple dimensions, while specialized policies may intentionally concentrate on a narrower administrative or technical mandate. A lower total PMC score therefore represents narrower structural coverage under a common analytical framework, not necessarily inferior policy design.

The text-mining results provide a complementary descriptive perspective. Management, information, data, service, health, and technology were among the most frequent terms in the corpus, which is broadly consistent with the prominence of governance, data resources, and service-related dimensions in the PMC analysis. However, term frequency and indicator coding capture different aspects of the texts. Frequency reflects the salience of vocabulary, whereas PMC coding records whether predefined structural elements are explicitly present. Their convergence therefore supports a coherent descriptive interpretation of the corpus without treating word frequency as an independent validation of the PMC scores.

Finally, the robustness analyses indicate that the principal dimension pattern is not driven by a single policy or by the closely related P11/P12/P13 policy family. X6 remained the highest and X7 the lowest dimension in all leave-one-policy-out runs, while family collapse produced only a small change in the mean PMC and a limited exchange between adjacent middle-ranked dimensions. The positive associations between each Xi and PMC excluding Xi further indicate that dimensions with greater coverage tend to co-occur with broader coverage elsewhere in the policy structure. These analyses support the stability of the observed configuration, but they should not be interpreted as external validation of policy quality or implementation performance.

### 4.2 Policy implications

The results suggest that policy refinement should focus less on maximizing the number of indicators in every document and more on strengthening functionally necessary links among specialized policies. Comprehensive strategies may reasonably be expected to articulate broader relationships among data, platforms, regulation, protection, innovation, services, and implementation support, whereas narrowly focused regulations or technical standards should be judged against the requirements of their own mandates.

The comparatively low and highly dispersed coverage of data protection nevertheless identifies an area where explicit cross-document coordination is particularly important. Policies that materially depend on personal, clinical, or administrative health data can more clearly specify security responsibilities, assessment procedures, monitoring and incident response, personal-information protection, and relevant infrastructure safeguards, or explicitly connect these obligations to binding instruments elsewhere in the policy system. Such cross-referencing can improve textual traceability without requiring every policy to reproduce the complete security framework.

Industrial-innovation provisions similarly benefit from clearer differentiation among technology R&D, intelligent-device deployment, industrial-ecosystem cultivation, pilot translation, and collaborative innovation. These elements need not appear in every specialized policy, but comprehensive strategies can clarify how technology development connects with standards, regulation, service delivery, and mechanisms for scaling successful pilots. More generally, the 48-indicator framework can be used as a diagnostic checklist for identifying necessary policy interfaces rather than as a requirement that every document maximize structural breadth.

### 4.3 Research implications

Methodologically, the study demonstrates the value of combining source locking, a fully specified domain-specific codebook, evidence-linked binary coding, independent reliability assessment, and robustness analysis within PMC research. These procedures address several reproducibility problems that can arise when policy corpora, indicator definitions, or coding rules are insufficiently documented.

The study also illustrates the importance of separating structural coverage from policy effectiveness. PMC scores are useful for comparing the configuration of observable textual elements, but they cannot determine whether those provisions were enforced, adequately resourced, accepted by stakeholders, or associated with improved health outcomes. Future studies can therefore extend the present framework by linking coded policy structures with implementation indicators, organizational evidence, stakeholder assessments, and longitudinal health or service outcomes.

### 4.4 Limitations

This study has several limitations. First, the PMC framework measures the structural coverage of policy texts rather than implementation fidelity, regulatory effectiveness, service quality, or health outcomes. Binary coding improves cross-policy comparability but does not capture the intensity, legal force, resource commitment, or internal coherence of individual provisions. Although all 1,872 coding decisions were individually reviewed and finalized by the primary researcher and the independently coded subset showed high inter-coder reliability, indicator-level coding inevitably retains an element of interpretive judgment.

Second, the corpus is limited to national-level Chinese policies and includes documents with different functions, administrative purposes, and issue periods. Specialized policies may appropriately omit dimensions outside their mandates, whereas comprehensive strategies have more opportunities to accumulate structural elements. Three eligible policies also remained outside the quantitative analysis because complete auditable source texts could not satisfy the predefined source-lock requirements, introducing a limited potential for selection bias.

Third, the text-mining results depend on segmentation, stop-word selection, paragraph boundaries, and display thresholds. The documented and deterministic preprocessing pipeline improves reproducibility but cannot eliminate all preprocessing sensitivity. Future research could extend the analysis to subnational policy systems and, more importantly, connect textual structural coverage with implementation data, stakeholder assessments, service utilization, and longitudinal outcomes.

## 5 Conclusions

Using a source-locked corpus of 39 national-level policies and a complete 48-indicator framework, this study assesses the structural coverage of China’s digital health management policy system. The independently coded subset showed high inter-coder reliability (96.78% agreement; Cohen’s κ = 0.914), supporting the reproducibility of the coding framework.

The mean PMC score was 6.654. Regulatory governance, standards and institutional arrangements, and data resources showed the most extensive average coverage, whereas data protection and industrial innovation were comparatively less and more unevenly represented. These patterns remained stable in leave-one-policy-out and policy-family sensitivity analyses.

The results should be interpreted as a map of policy-text configuration rather than a ranking of implementation success or policy effectiveness. Their main value lies in identifying which structural elements are consistently incorporated across the national policy system, which are distributed selectively across specialized instruments, and where stronger coordination among policies may improve the clarity and completeness of the overall governance architecture.

## Supporting information

S1 Data Final policy-by-indicator coding matrix and calculated PMC results. (XLSX)

S1 File Reproducible analysis scripts, stop-word list, and machine-readable outputs. (ZIP)

S2 Table Hypothetical 3%, 5%, and 10% bit-flip stress-test results, including rank correlation and category-retention statistics. (CSV)

S3 Table Policy identification, eligibility, and source-lock status. (XLSX)
